# High Fracture Risk of Femoral Bone Metastasis Treated with Palliative Radiotherapy in Recent Years

**DOI:** 10.3390/curroncol31120549

**Published:** 2024-11-22

**Authors:** Kenji Makita, Hidehiro Hojo, Hidekazu Oyoshi, Takeshi Fujisawa, Masaki Nakamura, Gyo Uchida, Yume Koike, Yuzheng Zhou, Kento Tomizawa, Keiko Fukushi, Sadamoto Zenda

**Affiliations:** 1 Department of Radiation Oncology, National Cancer Center Hospital East, Kashiwa 277-8577, Chiba, Japan; hhojo@east.ncc.go.jp (H.H.); hoyoshi@east.ncc.go.jp (H.O.); takfujis@east.ncc.go.jp (T.F.); masanaka@east.ncc.go.jp (M.N.); gyuchid@east.ncc.go.jp (G.U.); yukoi2@east.ncc.go.jp (Y.K.); ushuu@east.ncc.go.jp (Y.Z.); ktomizaw@east.ncc.go.jp (K.T.); kefukush@east.ncc.go.jp (K.F.); szenda@east.ncc.go.jp (S.Z.); 2 Department of Radiation Oncology, National Hospital Organization Shikoku Cancer Center, Matsuyama 791-0280, Ehime, Japan

**Keywords:** bone metastasis, femoral bone, palliative, radiotherapy, pathological fracture

## Abstract

Bone-modifying agents (BMAs) have been widely used to reduce skeletal-related events, including pathological fractures. Herein, we aimed to clarify the incidence of pathological fractures caused by high-risk femoral bone metastases after palliative radiotherapy (RT) in the BMA era and evaluate the necessity of prophylactic surgical stabilization. We assessed 90 patients with high-risk femoral bone metastases, indicated by Mirels’ scores ≥ 8, without pathological fractures and surgical fixations, who received palliative RT at our institution between January 2009 and December 2018. Pathological fracture incidence was analyzed using the Kaplan–Meier method and was 22.8% and 31.0% at 2 and 6 months, respectively. Pathological fractures were caused by 17 of 65 lesions (26.2%) and 9 of 25 lesions (36.0%) in patients who received BMAs and those who did not, respectively (*p* = 0.44). Additionally, 17 of 42 lesions (40.5%) and 9 of 48 lesions (18.8%) with axial cortical involvement ≥30 and <30 mm, respectively, caused pathological fractures (*p* = 0.02). The incidence of pathological fractures was high among patients with high-risk femoral bone metastases treated with palliative RT, particularly those with axial cortical involvement ≥30 mm. Therefore, aggressive indications for prophylactic surgical stabilization are warranted for high-risk femoral metastases despite BMA administration.

## 1. Introduction

Bone metastasis has been reported in approximately 50% of patients with cancer [1], and skeletal-related events (SREs) are common complications of bone metastasis. SREs include pathologic fracture, spinal cord compression, malignant hypercalcemia, palliative radiation to the bone, and palliative bone surgery. Recently, several studies have suggested that prophylactic radiotherapy (RT) reduces the rate of SREs and hospitalizations [2], resulting in improved prognosis [2,3,4]. Therefore, prophylactic RT for preventing SREs is an important treatment strategy.

Pathological fracture, which is a break in the bone caused by bone metastasis, is a major SRE. Since pathological fracture is a common problem in managing patients with bone metastasis, Mirels et al. proposed a scoring system for predicting pathological fracture caused by bone metastasis [5,6]. Currently, the scoring system, which is based on pain intensity, location, type (lytic, mixed, or blastic), and the amount of bone involvement, is widely used to predict the risk of pathological fractures after palliative RT. Based on the scoring system, femoral bone metastasis has been associated with the greatest risk of pathological fractures. Furthermore, lesions with scores ≥ 8 are considered high-risk lesions for pathological fractures and warrant prophylactic surgical stabilizations before palliative RT [6].

Recently, bone-modifying agents (BMAs), which are effective in reducing SREs, have been widely used to treat bone metastases [7,8,9]. Consequently, prophylactic surgical stabilizations before palliative RT may be omitted for these cases. Therefore, in this study, we aimed to clarify the incidence of pathological fractures in patients with high-risk femoral bone metastases following palliative RT in the BMA era.

## 2. Materials and Methods

### 2.1. Patients

Between January 2009 and January 2018, 144 patients with lesions (n = 158) who received palliative RT for femoral bone metastasis and had no pathological fractures and surgical fixation at our institution were enrolled in this study. Mirels’ scoring system was used to evaluate the risk of pathological fracture caused by bone metastasis. Patients with Mirels scores ≥ 8 were included in this retrospective analysis. The ethics committee of our institution approved this study (approval number: 2017-440), and the requirement for informed consent was waived.

### 2.2. Evaluation

The Mirels scoring system assesses pain intensity (mild, moderate, or functional), location (only the femur), type (lytic, mixed, or blastic), and amount of bony involvement (<1/3, 3/1–3/2, or >2/3) [4]. In this study, the only location of bone metastasis was the proximal femur. In addition to the factors considered by the Mirels’ scoring system, the effect of axial cortical involvement (≥30 or <30 mm) was evaluated using computed tomography images.

### 2.3. Bone-Modifying Agents

Zoledronic acid at 4 mg or Denosumab at 120 mg injection was administered every 3–4 weeks. All BMA administrations were initiated before or concurrently with palliative RT for femoral bone metastasis. In principle, patient wishes, general condition, or complications, including the risk of osteonecrosis of the jaw or renal impairment, contributed to whether or not the BMA was administered.

### 2.4. Statistical Analyses

Axial cortical involvement (≥30 or <30 mm) and BMA administration were assessed using the chi-square or Fisher’s exact test. Fracture incidence was analyzed using the Kaplan–Meier method. Time analysis was calculated from the first day of palliative RT. The patient’s death was considered the sensor. All statistical analyses were performed using R (The R Foundation for Statistical Computing, version 3.5.0, Vienna, Austria), which is designed to incorporate statistical functions frequently used in biostatistics.

## 3. Results

### 3.1. Clinical Characteristics

Ninety lesions from eighty-six patients with high-risk femoral bone metastases were evaluated in this retrospective analysis. The factors considered by the Mirels’ scoring system were pain intensity (mild, 42 lesions; moderate, 36 lesions; and functional, 12 lesions); type (lytic, 10 lesions; mixed, 70 lesions; and blastic, 10 lesions); and amount of bony involvement (<1/3, 34 lesions; 1/3–2/3, 30 lesions; and >2/3, 26 lesions). Patient characteristics are summarized in Table 1. BMAs were administered to 61 (70.9%) patients. Notably, Zoledronic acid and Denosumab were administered to 42 and 19 patients, respectively. The dose fractionations of palliative RT for femoral bone metastasis were 30 Gy in 10 fractions (45 lesions [50.0%]), 20 Gy in five fractions (27 lesions [30.0%]), and 8 Gy in a single fraction (18 lesions [20.0%]).

### 3.2. Risk of Pathological Fracture

The median follow-up duration for the censored patients was 83 days (range, 8–3566 days). At the last follow-up, 26 (28.9%) lesions caused pathological fractures at the index lesion sites (Table 2).

**Figure 1:**
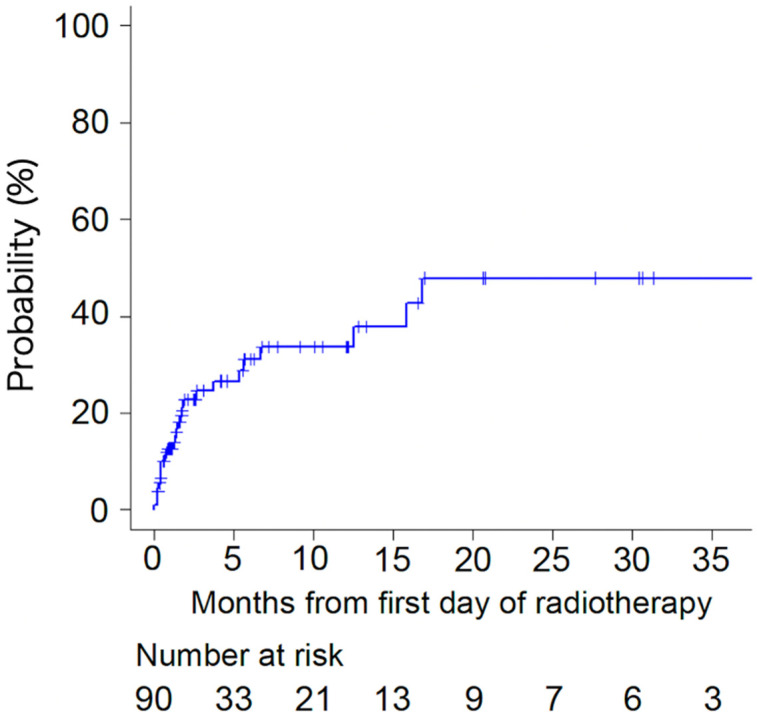
Incidence of pathological fracture.

The incidence of pathological fractures was 22.8% and 31.0% at 2 and 6 months, respectively (Figure 1). Pathological fractures were observed in 17 of 65 lesions (26.2%) and 9 of 25 lesions (36.0%) in patients who received BMAs and those who did not, respectively, with no significant difference (*p* = 0.44, Table 3). Among patients who received BMAs, pathological fractures were caused by 11 of 44 lesions (25%) and 6 of 21 lesions (29%) in patients who received Zoledronic acid and Denosumab, respectively, with no significant difference (*p* = 0.77). However, 17 of 42 lesions (40.5%) and 9 of 48 lesions (18.8%) with axial cortical involvement ≥30 and <30 mm, respectively, caused pathological fractures (*p* = 0.02, Table 3).

Pathological fractures were caused by 15 of 65 lesions (23.1%) and 11 of 35 lesions (31.4%) in patients with lung or breast cancer and those with other primary tumors, respectively, with no significant difference (*p* = 0.81, Table 3). Furthermore, pathological fractures were caused by 3 of 18 lesions (16.7%) and 23 of 72 lesions (31.94%) in patients treated with 8 Gy in a single fraction and those treated with 20 Gy in 5 fractions or 30 Gy in 10 fractions, respectively, with no significant difference (*p* = 0.25, Table 3).

## 4. Discussion

In the present study, we investigated the risk of pathological fracture in patients with high-risk femoral bone metastases, indicated by a Mirels’ score ≥ 8, treated with palliative RT. We found that the incidence of pathological fractures remained high in patients with high-risk femoral bone metastases treated with a combination of palliative RT and BMAs. Notably, the femoral bone metastasis with axial cortical involvement ≥30 mm was associated with a high risk of pathological fracture.

Numerous studies have reported a 5–40% incidence of pathological fractures among patients with femoral bone metastases treated with palliative RT [6,10,11,12]. However, most studies did not evaluate the risk of pathological fractures in the BMA era, where they have shown that SREs, including pathological fractures, can be prevented or delayed using BMAs [13,14]. Furthermore, a combination of BMA and palliative RT reportedly improves local control of irradiated bone metastases [15,16,17]. Notably, several animal studies have suggested that a combination of BMA and RT restores bone quality and reduces fracture risk [18,19,20,21]. However, in the present study, the improvement in the incidence of pathological fractures was lesser in patients with high-risk femoral bone metastasis using a combination of palliative RT and BMA than in those using palliative RT alone (BMAs + RT vs. RT alone: 26.2% vs. 36.0%). Therefore, the effectiveness of BMA in preventing pathological fractures in patients with high-risk femoral bone metastasis could not be confirmed. Consequently, additional local treatments (surgical stabilization is preferable to palliative RT alone) should be performed for high-risk femoral bone metastasis, along with BMA administration.

Mirels’ scoring system is commonly used to predict the risk of pathological fracture caused by bone metastasis [4]. In contrast, the axial cortical involvement of the lesion ≥30 mm is considered a high-risk factor for fracture based on previous studies that reviewed femoral bone metastasis within the multicenter prospectively randomized Dutch Bone Metastasis Study or a validation study that confirmed the use of 30 mm threshold for axial cortical involvement to assess fracture risk [11,22,23]. Similarly, we found that axial cortical involvement of the lesion ≥30 mm was a high-risk factor (≥30 mm vs. <30 mm: 40.5% vs. 18.8%) for femoral bone fracture. Therefore, lesions should be evaluated for axial cortical involvement (≥30 mm vs. <30 mm) in addition to using the Mirels’ scoring system. Furthermore, the combination of palliative RT with BMA was not adequate to prevent pathological fractures in these cases. Therefore, aggressive surgical stabilizations, such as intramedullary nailing, hemiarthroplasty or total hip arthroplasty, and endoprosthetic reconstructions, are required for cases of axial cortical involvement of the lesion ≥ 30 mm, as surgical fixation for pain control and mobilization is useful in patients with femoral bone metastasis [24,25].

This study has some limitations owing to its retrospective nature. First, the sample size was small, which reduced the statistical power. Second, heterogeneous primary tumors were included in this study. Different primary tumors have different radiosensitivities, which may have affected the incidence of pathological fractures caused by high-risk femoral bone metastasis treated with palliative RT in this study. Third, various studies have shown differences in the efficacy of different types of BMA in preventing SREs [26,27,28,29,30]. Therefore, the type of BMA administered may have also influenced the incidence of pathological fractures in patients with high-risk femoral bone metastasis. Fourth, the reason for omitting prophylactic surgical stabilizations was unclear in many patients. However, in some individuals, patient wishes, general conditions, or complications contributed to whether or not the surgical stabilizations were performed. Similarly, the reasons why BMA was not administered to some patients were unclear in all patients with Mirels’ score ≥8. This suggested a potential selection bias associated with receiving BMA or not. Finally, only a few patients were treated with 8 Gy, a single fraction, which was insufficient to assess fracture risk based on the RT dose. Consequently, large prospective studies are required in the future to address these limitations.

## 5. Conclusions

The incidence of pathological fractures remained high in patients who received palliative RT for femoral bone metastasis with Mirels scores ≥ 8 despite BMA administration. Therefore, aggressive indications of prophylactic surgical stabilizations are warranted even when a combination of palliative RT with BMA is used, particularly in cases with axial cortical involvement of the lesion ≥ 30 mm.

## Figures and Tables

**Table 1:** Characteristics of the 90 lesions.

Characteristic		No. of Lesions
Age (years)		33–82 (median 64)
Sex	Female	41 (46%)
	Male	49 (54%)
Primary tumor	Lung	42 (47%)
	Breast	13 (14%)
	Esophagus	10 (11%)
	Renal	5 (6%)
	Pancreatic	4 (4%)
	Liver	3 (3%)
	Large intestine	3 (3%)
	Prostate	2 (2%)
	Uterus	2 (2%)
	Stomach	2 (2%)
	Other	4 (4%)
Mirels’ scores	8 points	37 (41%)
	9 points	53 (59%)
Cortical involvement	≥30 mm	42 (47%)
	<30 mm	48 (53%)
BMA administration	Yes	65 (72%)
	No	25 (28%)
Chemotherapy	Yes	51 (57%)
	No	39 (43%)
Opioid use	Yes	51 (57%)
	No	39 (43%)
Opioid (daily oral morphine equivalent; mg)		10 (0–120)
Fractionation schema	8 Gy in 1 fraction	18 (20%)
	20 Gy in 5 fractions	27 (30%)
	30 Gy in 10 fractions	45 (50%)

BMA, bone-modifying agent.

**Table 2:** Characteristics of the 26 lesions that caused pathological fractures.

Characteristic		No. of Lesions
Age (years)		33–82 (median 64)
Sex	Female	12 (46%)
	Male	14 (54%)
Primary tumor	Lung	12 (46%)
	Breast	3 (12%)
	Esophagus	1 (4%)
	Renal	3 (12%)
	Pancreatic	1 (4%)
	Liver	2 (8%)
	Large intestine	1 (4%)
	Prostate	1 (4%)
	Uterus	1 (4%)
	Stomach	0 (0%)
	Other	0 (0%)
Mirels’ scores	8 points	7 (27%)
	9 points	19 (73%)
Cortical involvement	≥30 mm	17 (65%)
	<30 mm	9 (35%)
BMA administration	Yes	17 (65%)
	No	9 (35%)
Opioid use	Yes	14 (54%)
	No	12 (46%)
Opioid (daily oral morphine equivalent; mg)		15 (0–30)
Fractionation schema	8 Gy in 1 fraction	3 (12%)
	20 Gy in 5 fractions	9 (35%)
	30 Gy in 10 fractions	14 (54%)

BMA, bone-modifying agent.

**Table 3:** Comparison of characteristics between groups with and without fracture.

	Total (n = 90)	Fracture (n = 26)	No Fracture (n = 64)	*p*-Value
Age (years)	33–82 (median 64)	37–82 (median 64)	33–82 (median 65)	0.39
≥65	44 (49%)	12 (46%)	32 (50%)	0.82
<65	46 (51%)	14 (54%)	32 (50%)	
Sex				
Female	41 (46%)	12 (46%)	29 (45%)	>0.99
Male	49 (54%)	14 (54%)	35 (55%)	
Primary tumor				
Lung or breast	55 (%)	15 (58%)	40 (63%)	0.81
Others	35	11 (42%)	24 (37%)	
Mirels’ scores				
8 points	37 (41%)	7 (27%)	30 (47%)	0.10
9 points	53 (59%)	19 (73%)	34 (53%)	
Cortical involvement				
≥30 mm	42 (47%)	17 (65%)	25 (39%)	0.02
<30 mm	48 (53%)	9 (35%)	39 (61%)	
BMA administration				
Yes	65 (72%)	17 (65%)	48 (75%)	0.44
No	25 (28%)	9 (35%)	16 (25%)	
Opioid administration				
Yes	51 (57%)	14 (54%)	37 (58%)	0.82
No	39 (43%)	12 (46%)	27 (42%)	
Radiotherapy schedule				
8 Gy/1 fr	18 (20%)	3 (12%)	15 (23%)	0.25
20 Gy/5 fr or 30 Gy/10 fr	72 (80%)	23 (88%)	49 (77%)	

BMA, bone-modifying agent.

## Data Availability

No new data were created or analyzed in this study. Data are contained within the article.
